# Association of CETP *Taq1B* and -*629C* > *A* polymorphisms with coronary artery disease and lipid levels in the multi-ethnic Singaporean population

**DOI:** 10.1186/1476-511X-12-85

**Published:** 2013-06-08

**Authors:** Yongjian Lu, Naeimeh Tayebi, Hongzhe Li, Nilmani Saha, Hongyuan Yang, Chew-Kiat Heng

**Affiliations:** 1 Department of Paediatrics, Yong Loo Lin School of Medicine, National University of Singapore, Singapore, Singapore; 2 Department of Biochemistry, National University of Singapore, Singapore, Singapore; 3 School of Biotechnology and Biomolecular Sciences, The University of New South Wales, Sydney 2052, Australia

**Keywords:** Cholesteryl ester transfer protein, -*629C* > *A* polymorphism, *TaqB1* polymorphism, HDL-cholesterol, Coronary artery disease

## Abstract

**Background:**

Hyperlipidaemia is a major risk factor for coronary artery disease (CAD) and cholesteryl ester transfer protein (CETP) gene polymorphisms are known to be associated with lipid profiles.

**Methods:**

In this study, we investigated the association of two polymorphisms in the CETP, *Taq1B* (rs708272) and -*629C* > *A* (rs1800775), with CAD and lipid levels HDL-C in 662 CAD + cases and 927 controls from the Singapore population comprising Chinese, Malays and Indians.

**Results:**

*TaqB2* frequency was significantly lowest in the Malays (0.43) followed by Chinese (0.47) and highest in the Indians (0.56) in the controls. The *B2* allele frequency was significantly lower in the Chinese CAD + cases compared to the controls (p = 0.002). The absence of the *B2* allele was associated with CAD with an OR 2.0 (95% CI 1.2 to 3.4) after adjustment for the confounding effects of age, smoking, BMI, gender, hypertension, dyslipidemia and diabetes mellitus. The *B2* allele was significantly associated with higher plasma HDL-C levels in the Chinese men after adjusting for confounders. Associations with plasma apoA1 levels were significant only in the Chinese men for *Taq1B* and -*629C* > *A*. In addition, the Taq1B polymorphism was only associated with plasma Apo B and Lp(a) in the Malay men. Significant associations were only found in non-smoking subjects with BMI <50^th^ percentile. In this study, the LD coefficients between the *Taq1B* and -*629C* > *A* polymorphisms seemed to be weak.

**Conclusion:**

The absence the *Taq1B2* allele was associated with CAD in the Chinese population only and the minor allele of the Taq1B polymorphism of the *CETP* gene was significantly associated with higher plasma HDL-C levels in Chinese men.

## Introduction

The inverse relationship between plasma high density lipoprotein-cholesterol (HDL-C) and the incidence of coronary artery disease (CAD) is well established [1,2]. HDL is thus believed to be a protective factor against CAD. The removal of cholesterol from tissues and its delivery to the liver for excretion constitute the process of reverse transport [3,4]. One of the important components of this process is the cholesteryl ester transfer protein (CETP). CETP facilitates the transfer of cholesteryl ester from HDL to triglyceride-rich lipoproteins in exchange for triglycerides (TG) [5,6]. Many CETP DNA polymorphisms that may alter the function of CETP have been reported [7-9]. However, the association of these polymorphisms with phenotypes such as lipid levels and the CAD remain inconsistent [10-14].

Among the several *CETP* polymorphisms reported, some are found to be associated with plasma levels of HDL-C. One of the most widely studied polymorphic sites on the *CETP* gene is the *Taq1B* site [15,16]. This is a polymorphism at nucleotide 277 in intron 1(277C > T, rs708272). However, the *B2* allele of this polymorphism has been shown to be associated with elevated plasma HDL-C levels in numerous studies [17-21]. It has been reported that the *Taq1B* site is in linkage disequilibrium (LD) with another polymorphic site (−*629C* > *A*, rs1800775) in the CETP promoter region [22,23] and the latter could explain the association between *Taq1B* and HDL-C levels since it was shown to be a functional base substitution. However, a subsequent study in the Icelandic population reported that the -*629C* > *A* polymorphism does not explain the association of *Taq1B* polymorphism with risk of myocardial infarction although both are associated with HDL-C levels [24].

The effects of CETP polymorphisms have been reported in some of the Asian populations such as Sri Lankans [25],Koreans [26],Taiwanese [27,28] Chinese [29], Indians [30,31] and Turkish [32].The multi-ethnic Singaporean population is also studied for the *Taq1B* and -*629C* > *A* polymorphisms at the CETP locus for their association with lipid levels [33]. However, the study was conducted in the general population and hence the association of the polymorphisms with CAD could not be determined. On the other hand it is noted that the mortality rate of CAD in the ethnic Asian Indians is more than three times of the Chinese [34]. The impact of the CETP genetic variants on complex outcomes such as CAD is also warranted by Thompson et al. [8]. As far as lipid profile is concerned the plasma HDL-C level is shown to be one of the most discriminative factors for CAD in Singapore population [35].

Singapore offers a good context for studying the genetic effects on CAD and lipid levels as three major diverse ethnic groups are residing in this small island state. We report here the allele frequencies and the association of the CETP *Taq1B* and -*629C* > *A* polymorphisms with CAD and lipid levels in the Chinese, Malays and Asian Indians residing in Singapore.

## Materials and methods

### Ethics

This study was approved by the institutional ethics committee of National University of Singapore (NUS) IRB and all subjects gave their informed consent for their participation in the study.

### Study design

This was a case–control study. The cases were the angiographically confirmed CAD patients while the controls were selected from the general population who attended routine medical examination at a health screening centre as required by their employers.

### Study subjects

A total of 662 angiographically confirmed CAD patients (CAD+) who were admitted consecutively for coronary artery by-pass graft at Singapore’s National Heart Centre between 1995 and 2002 were included as cases in this study. All patients had more than 50% stenosis in at least one of the major coronary arteries as diagnosed by angiography.

Separately, 927 consecutive individuals from the general population, who attended routine medical examination at a community screening centre, without the history of heart disorders and ECG abnormalities were recruited as controls. 

### Sample collection and biochemical tests

The blood samples from the CAD + cases were collected before their by-pass operation for routine examination including lipid levels. The blood samples from the controls were collected in tubes containing EDTA as anti-coagulant after an overnight fast of at least 10 hours as a part of routine examinations and tests. The plasma was separated by centrifugation and aliquot portions were stored at −20°C until use. Measurement of plasma lipids and DNA extraction were carried out as previously described [36].

### Genotyping

The *Taq1B* polymorphism was genotyped by restriction fragment length polymorphism (RFLP) assay. A 535 bp fragment in the intron 1 of the CETP gene was amplified by using the primers 5' CACTAGCCCAGAGAGAGGAGTGCC3' and 5' CTGAGCCCAGCCGCACACTAAC3'. Amplification was carried out in a volume of 20 μl containing 1 - 10μg of genomic DNA, 20 pmol of each primer, 0.5 mM of each dNTP, reaction buffer, 2% DMSO and 1.0 U of Taq DNA polymerase. Amplified products were digested overnight with 8 μl of *Taq*1 at 65 ^0^C. The resulting fragments were 174 and 361 bp for *B1* allele and 535 bp for *B2* allele.

Allele specific amplification was used to identify the -*629C* > *A* promoter polymorphism. The common sense primer was GGCAGCTTTGGTATTGGAG. The specific anti-sense primers for the *A* and *C* allele were GATATGCATAAAATAACTCTG*A*GG and GATATGCATAAAATAACTCTG*A*GT respectively. We found that creating a deliberate mismatch at the -627C nucleotide by changing it to *A* in both primers could yield better allele discrimination. The amplicons were visualized by electrophoresis on 2.5% agarose gel. The specific amplified fragment was 398 bp in length. Another pair of primers from ABCA1 gene was introduced into the system as an amplification control: sense, CTTCACTCCCATATTTCAGAACTTG and anti-sense, ATCTCCATTAAAGCATCCTACAGC. The control product was 276 bp long.

### Statistical analysis

The majority of statistical analysis was performed using SPSS Version 19. The chi-squared (χ^2^) analysis was used to test for departure from Hardy-Weinberg equilibrium (HWE) and for differences in allele frequencies between groups. Analysis of covariance (ANCOVA) was performed to determine the effects of the *CETP* gene polymorphisms on the various lipid traits using significant confounding factors such as age, BMI and cigarette smoking as covariates. The significance of the sample variance was tested by *F* and *P* values while percentage of explained variance (*R*^*2*^ x 100) was calculated from the sum of squares. Due to their skewed distribution, the raw data for TG and Lp(a) were normalized by natural logarithm transformation prior to analysis. The LD between the *Taq1B* and -*629C* > *A* sites was indicated by *r*^*2*^. Statistical significance was taken at α =0.05.

In the combined samples, the association analysis was performed between plasma HDL-C levels and *CETP* gene polymorphisms using linear model and was also assessed for the homogeneity using Cochrane's *Q* statistic and I^2^ heterogeneity index in meta-analysis in *PLINK*.

Wherever appropriate, the analyses were performed separately for men and women given the differences in lipid levels that exist in these groups. The association between *CETP* polymorphism and lipid profile was evaluated only in the control group because the lipid levels in the CAD + cases may be influenced by the lipid lowering agents that some of these patients had received following the diagnosis of CAD.

## Results

### Subject characteristics

The subject characteristics of the study population for three ethnic populations for CAD + and controls are given in Table 1.

**Table 1:** Demographics of the study subjects in three ethnic populations

	**Chinese**			**Malays**			**Indians**		
**Variables**	**Control**	**CAD+**	***P***	**Control**	**CAD+**	***P***	**Control**	**CAD+**	***P***
	**n = 383**	**n = 442**		***n *=155**	***n*** **= 110**		***n*** **= 389**	***n*** **= 110**	
Age (years)	42.74 ± 14.2	59.34 ± 8.9	<0.0005	40.66 ± 9.24	59.08 ± 9.18	<0.0005	42.43 ± 14.08	60.35 ± 10.25	<0.0005
BMI (kg/m^2^)	23 ± 3.61	24.24 ± 3.67	ns	25.17 ± 4.06	26.14 ± 3.62	ns	24.77 ± 4.8	24.9 ± 3.2	ns
Smokers* (%)	17.4	52.9	<0.0005	52.7	49	ns	13.6	43.8	<0.0005
Sex (%)									
Female	46.1	21.9	<0.0005	8.7	23.9	0.001	37.8	16.5	<0.0005
Male	53.9	78.1		91.3	76.1		62.2	83.5	
Dyslipidemia	46.3%	32.6%	<0.0005	56.4%	27.5%	<0.0005	63.4%	21.1%	<0.0005
Hypertension	8.2%	69.1%		4.1%	72.7%		9.8%	61.8%	
Diabetes Mellitus	2.7%	43.3%		3.4%	62.6%		6.4%	61.8%	
TC(mM)	5.81±1.19	4.57±1.14	<0.0005	5.85±1.22	4.6±1.13	<0.0005	5.61±1.22	4.25±1.99	<0.0005
HDL-C (mM)	1.4 ± 0.4	0.97 ± 0.26	<0.0005	1.19 ± 0.27	0.91 ± 0.27	<0.0005	1.11 ± 0.32	0.88 ± 0.23	<0.0005
LDL-C (mM)	3.57 ± 1.17	2.78 ± 0.99	<0.0005	3.7 ± 1.23	2.89 ± 1.03	<0.0005	3.62 ± 1.15	2.58 ± 0.86	<0.0005
TG (mM)	1.84 ± 1.9	1.37 ± 0.66	0.008	2.09 ± 1.31	1.51 ± 0.68	0.014	1.93 ± 1.45	1.29 ± 0.63	<0.0005
ApoA1 (mg/dl)	144.4 ± 22.04	119.47 ± 20.5	<0.0005	129.29 ± 18.5	115.29 ± 20.8	<0.0005	135.85 ± 25.11	110.01 ± 19.8	<0.0005
ApoB (mg/dl)	106.2 ± 31.4	89.78 ± 22.6	<0.0005	121.36 ± 29	103.64 ± 28	<0.0005	122.87 ± 33.04	102.3 ± 26.5	<0.0005
Lp(a) (mg/dl)	16.90 ± 22.67	24.33 ± 27.38	0.007	14.66 ± 15.16	19.5 ± 16.28	0.032	21.9 ± 21.17	26.89 ± 11.4	0.038

Age, BMI and plasma lipid profiles are presented in mean ± SD. Continues and categorical data were tested using independent sample *t*-test and Chi-square test respectively.

TC, total cholesterol; HDL-C, high-density lipoprotein cholesterol; LDL-C, low-density lipoprotein cholesterol; TG, triglyceride; BMI, body mass index; Apo, Apolipoprotein; Lp(a), lipoprotein(a).

*Smokers and ex-smokers form one category while non-smokers constitute another.

Significant differences between CAD + cases and controls were observed for most components of the lipid profiles. Among these, TC, LDL-C, TG and apolipoprotein (apo) B were significantly lower in the CAD + cases as against the controls of all ethnic groups. Proportion of subjects with Hypertension and diabetes was significantly higher in CAD + cases as compared to control. However, higher proportion of subjects was dislipedemic in controls as compare to cases. These were expected as most CAD + cases were on lipid-lowering drugs. Plasma levels of Lp(a), which is very resistant to modification, was significantly higher in the CAD + cases as against the controls in all ethnic groups . As the mean age of the CAD + cases was significantly higher than the controls, we carried out logistic regression to determine if age was significantly related to the allele frequencies of both polymorphisms and found that it was not. We were therefore confident that any significant differences in allele frequencies between CAD + cases and controls were not due to the confounding effects of age.

### Frequency distribution of the polymorphisms

**Figure 1:**
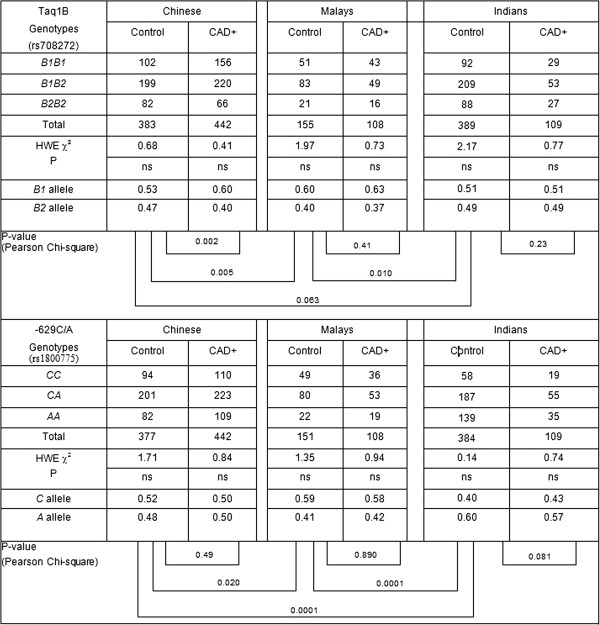
**Genotype and allele frequencies of the *TaqIB *and *-629C/A *promoter polymorphisms in the three ethnic populations for CAD+ cases and controls.** HWE χ^2^ : χ^2^ -test for departure from Hardy Weinberg equilibrium. Pearson Chi-Square is the test statistic for allele frequency comparison between controls and CAD+ cases. P-values were obtained after adjustment for gender, age, smoking status, body mass index, hypertension, dyslipidemia and diabetes mellitus.

Figure 1 shows the genotype and allele distributions of the two polymorphisms in the controls and CAD + cases, among the three ethnic groups in Singapore.

Based on frequencies in the controls, the *B2* allele frequency was lowest in the Malays (0.43) followed by Chinese (0.47) and highest in the Indians (0.56). The *B2* frequency was significantly lower in Malay controls as compared to the Indians (p = 0.01) and Chinese (p = 0.005) controls. In all ethnic groups, *B1* was the major allele.

The -*629C* allele was predominant in Chinese (0.52) and Malays (0.60) controls, but not in Indians (0.30).

Allele frequencies significantly varied between the controls of all ethnic groups. The genotype distributions of both polymorphisms in all three ethnic groups were consistent with Hardy Weinberg expectations.

### Case–control differences in allele frequencies

Apart from the highly significant lower *B2* allele in the Chinese CAD + cases compared to the controls (p = 0.002), the allele frequencies for both the promoter and *Taq1B* polymorphisms are not significantly different between CAD + cases and controls in all the ethnic groups.

The odds ratio for CAD associated with the absence of *B2* allele is 2.0 (95% CI 1.2 to 3.4) after adjustments were made for the confounding effects of age, cigarette smoking, BMI, gender, hypertension, dyslipidemia and diabetes mellitus by logistic regression. The absence of *B2* allele is hence independently associated with CAD in the Chinese but not for the Malays and Indians. There is no significant association of the -*629C* > *A* polymorphism with CAD.

### LD between Taq1B and-629C > A sites

The LD coefficients between the *Taq1B* and -*629C* > *A* polymorphisms are presented in Table 2. It is interesting to note that LD is stronger in control than in cases for the Chinese (0.38 vs. 0.10) and Malays (0.38 vs. 0.07), but not for the Indians (0.11 vs. 0.12). With the maximum r^2^ value in controls being 0.388, considering the cut off of 0.80 [37], there seems to be a weak LD between the two polymorphism studied.

**Table 2:** **Linkage disequilibrium coefficients between the *Taq1B *and** –***629C***/***A *sites in the three ethnic populations for CAD+ cases and controls**

		***r***^***2***^
	Chinese	0.380
Controls	Malays	0.388
	Indians	0.113
	Chinese	0.101
CAD+	Malays	0.074
	Indians	0.128

#### Impact of *CETP* polymorphisms on plasma lipid profile

The association of the *Taq1B* and -*629C* > *A* polymorphisms with lipid levels in our population show ethnic and gender-specificity (Tables 3 and 4). All analyses were carried out with age, BMI, smoking status, hypertension and diabetes mellitus as covariates. Consistent association of the minor allele *B2* with higher plasma HDL-C levels in the Chinese men was observed in the Taq1B polymorphism studied. Plasma HDL–C level was in the order *B1B1* < *B1B2* < *B2B2* (p = 0.004). Associations with plasma apoA1 levels were significant only in the Chinese men for *Taq1B* (p < 0.001) and -*629C* > *A* (p = 0.029). The other lipids that are significantly associated with the polymorphisms are shown in Tables 3 and 4.

**Table 3:** **Genotypic lipid levels** (**Mean ± SD**) **of the *Taq1B *polymorphism in the three ethnic populations for controls**

**Men**												
**n = 561**												
	**Chinese**				**Malays**				**Indians**			
	**n = 184**				**n = 138**				**n = 239**			
**Variables**	***B1B1***	***B1B2***	***B2B2***	***P***	***B1B1***	***B1B2***	***B2B2***	***P***	***B1B1***	***B1B2***	***B2B2***	***P***
	***n*** **= 50**	***n*** **= 95**	***n*** **= 39**	***R***^***2***^**x100**	***n*** **= 46**	***n*** **= 75**	***n*** **= 17**	***R***^***2***^**x100**	***n*** **= 54**	***n*** **= 133**	***n*=52**	***R***^***2***^**x100**
TC(mM)	5.62±1.12	5.88±1.25	6.03±1.24	P = 0.351	5.94±1.09	5.74±1.22	6.57±1.52	P = 0.055	5.81±1.16	5.90±1.29	5.62±1.30	P = 0.537
				1.14				3.908				0.51
LDL-C(mM)	4.04±0.93	4.13±1.07	4.33±1.21	P = 0.458	4.37±1.05	4.13±1.22	4.93±1.56	P = 0.051	4.19±1.22	4.32±1.28	4.11±1.32	P = 0.716
				0.85				3.99				0.27
HDL-C(mM)	1.15±0.23	1.28±0.35	1.31±0.32	**P** = **0**.**004**	1.16±0.31	1.16±0.24	1.23±0.20	P = 0.407	0.96±0.22	1.03±0.29	1.07±0.27	P = 0.101
				5.06				0.099				1.924
lnTG	2.17±2.59	2.33±2.96	1.93±0.63	P = 0.847	2.03±1.30	2.31±1.38	2.06±0.99	P = 0.569	2.31±1.24	2.34±1.75	1.85±1.20	P = 0.145
				0.169				0.756				1.613
TG (mM)	8.79	10.26	6.88		7.60	10.06	7.86		10.06	10.34	6.33	
ApoA-1(mg/dl)	130.96±12.93	142.21±19.14	140.81±21.43	**P** < **0**.**001**	125.65±20.87	127.93±16.78	130.79±13.39	P = 0.625	126.81±21.67	133.89±23.88	136.85±22.24	P = 0.109
				7.057				0.665				1.863
ApoB(mg/dl)	105.91±26.25	113.99±29.63	118.42±27.44	P = 0.191	127.65±26.95	118.54±29.96	105.67±26.69	**P** = **0**.**025**	130.61±32.71	130.66±32.95	124.02±33.58	P = 0.590
				1.690				3.992				0.435
lnLp(a)	1.82±0.71	2.11±1.09	2.04±0.84	P = 0.423	2.16±0.96	2.38±0.78	1.61±0.86	**P** = **0**.**026**	2.63±0.90	2.46±1.00	2.38±1.08	P = 0.467
				1.052				9.029				0.683
Lp(a)(mg/dl)	6.17	8.21	7.67		8.68	10.82	4.99		13.88	11.81	10.82	
**Women**												
**n = 316**												
	**Chinese**				**Malays**				**Indians**			
	**n = 162**				**n = 12**				**n = 142**			
**Variables**	***B1B1***	***B1B2***	***B2B2***	***P***	***B1B1***	***B1B2***	***B2B2***	***P***	***B1B1***	***B1B2***	***B2B2***	***P***
	***n*** **= 45**	***n*** **= 81**	***n*** **= 36**	***R***^***2***^**x100**	***n*** **= 5**	***n*** **= 5**	***n*** **= 2**	***R***^***2***^**x100**	***n*** **= 37**	***n*** **= 73**	***n*** **= 32**	***R***^***2***^**x100**
TC(mM)	6.06±1.10	5.51±0.97	5.56±1.25	P = 0.102	5.99±1.42	4.80±0.63	5.92±0.40	P = 0.260	5.23±1.13	5.23±1.15	5.53±1.17	P = 0.338
				3.536				30.203				1.304
LDL-C(mM)	4.22±1.18	3.70±0.96	3.62±1.27	P = 0.052	4.36±1.39	3.21±0.54	4.17±0.27	P = 0.223	3.56±1.18	3.58±1.17	3.80±1.17	P = 0.524
				3.132				29.203				0.765
HDL-C(mM)	1.56±0.37	1.53±0.45	1.68±0.42	P = 0.203	1.45±0.16	1.35±0.49	1.38±0.18	P = 0.972	1.20±0.365	1.24±0.34	1.41±0.33	P = 0.055
				1.736				0.006				4.272
lnTG	1.41±0.74	1.41±0.84	1.25±0.75	P = 0.733	0.89±0.33	1.24±1.01	1.86±1.53	P = 0.203	1.54±1.13	1.55±1.32	1.20±0.62	P = 0.291
				0.326				20.65				1.681
TG (mM)	4.11	4.11	3.50		2.43	3.44	6.42		4.67	4.71	3.31	
ApoA-1(mg/dl)	147.24±20.68	150.59±25.73	152.88±21.93	P = 0.561	144.13±11.75	146.33±23.79	149.33±8.50	P = 0.541	136.50±23.30	139.35±26.63	150.97±28.83	P = 0.07
				0.709				5.537				3.773
ApoB(mg/dl)	96.98±25.75	89.72±21.93	88.75±32.74	P = 0.476	99.80±26.75	95.40±10.45	109.00±2.83	P = 0.459	113.78±32.03	110.51±31.06	117.35±33.01	P = 0.596
				0.900				14.32				0.57
lnLp(a)	2.30±1.19	2.43±1.03	2.65±1.06	P = 0.428	2.31±0.77	2.82±0.88	3.22±1.001	P = 0.422	2.66±0.82	2.76±0.92	2.82±1.02	P = 0.841
				1.259				17.70				0.284
Lp(a)(mg/dl)	10.02	11.43	14.18	-	10.13	16.81	25.05	-	14.33	15.78	16.81	-

**Table 4:** **Genotypic lipid levels** (**Mean ± SD**) **of the** -***629C***/***A *promoter polymorphism in the three ethnic populations for controls**

**Men**												
**n = 574**												
	**Chinese**				**Malays**				**Indians**			
	**n = 201**				**n = 138**				**n = 235**			
**Variables**	***CC***	***CA***	***AA***	***P***	***CC***	***CA***	***AA***	***P***	***CC***	***CA***	***AA***	***P***
	***n*** **= 49**	***n*** **= 106**	***n*** **= 46**	***R***^***2***^**x100**	***n*** **= 43**	***n*** **= 74**	***n*** **= 21**	***R***^***2***^**x100**	***n*** **= 32**	***n*** **= 122**	***n *=81**	***R***^***2***^**x100**
TC (mM)	5.58±1.13	5.88±1.22	6.08±1.28	P = 0.094	5.74±1.05	5.97±1.42	5.96±0.89	P = 0.809	5.69±0.99	5.96±1.29	5.63±1.21	P = 0.270
				2.310				0.296				1.101
LDL-C(mM)	3.51±1.07	3.76±1.09	3.77±1.15	P = 0.370	3.68±0.95	3.76±1.46	3.74±1.05	P = 0.946	3.74±0.87	3.92±1.22	3.63±1.11	P = 0.354
				1.000				0.083				0.872
HDL-C(mM)	1.21±0.26	1.25±0.32	1.35±0.33	*P* = *0*.*114*	1.17±0.29	1.15±0.25	1.26±0.25	P = 0.433	1.02±0.37	0.99±0.24	1.07±0.27	P = 0.130
				3.280				0.996				1.746
lnTG	0.53±0.48	0.57±0.45	0.63±0.45	P = 0.736	0.55±0.52	0.69±0.55	0.59±0.54	P = 0.614	0.54±0.58	0.72±0.48	0.55±0.55	*P* = *0*.*087*
				0.286				0.621				2.017
TG (mM)	1.70	1.77	1.88	-	1.73	1.99	1.80	-	1.72	2.05	1.73	-
ApoA-1(mg/dl)	133.57±14.17	141.81±18.48	140.89±22.03	***P*** = ***0***.***029***	124.50±19.38	127.78±17.14	134.48±16.39	P = 0.114	133.94±28.71	129.91±22.68	135.95±22.59	P = 0.201
				3.350				3.238				1.409
ApoB(mg/dl)	111.43±30.09	116.13±28.86	120.56±32.60	P = 0.522	123.71±25.82	124.21±33.83	121.86±22.88	P = 0.850	124.41±28.28	134.53±33.71	122.61±31.47	*P* = *0*.*117*
				0.581				0.206				1.809
lnLp(a)	2.06±1.00	2.14±0.97	1.77±0.95	0.218	2.13±0.99	2.19±0.92	2.03±0.71	P = 0.608	2.73±0.87	2.46±1.12	2.50±1.05	P = 0.524
				1.865				1.095				0.722
Lp(a)(mg/dl)	7.84	8.49	5.89		8.39	8.97	7.58	-	15.28	11.68	12.07	-
**Women**												
**n = 331**												
	**Chinese**				**Malays**				**Indians**			
	**n = 173**				**n = 13**				**n = 145**			
**Variables**	***CC***	***CA***	***AA***	***P***	***CC***	***CA***	***AA***	***P***	***CC***	***CA***	***AA***	***P***
	***n*** **= 44**	***n*** **= 94**	***n*** **= 35**	***R***^***2***^**x100**	***n*** **= 6**	***n*** **= 6**	***n*** **= 1**	***R***^***2***^**x100**	***n*** **= 26**	***n*** **= 63**	***n*** **= 56**	***R***^***2***^**x100**
TC (mM)	5.83±1.01	5.75±1.18	5.51±1.05	P = 0.583	5.78±1.37	5.32±0.81	4.85±0.00	P = 0.665	5.32±1.25	5.16±1.03	5.42±1.21	P = 0.104
				0.562				7.841				2.776
LDL-C(mM)	3.62±1.01	3.57±1.18	3.21±1.04	P = 0.267	3.80±1.54	3.40±0.44	2.66±0.00	P = 0.620	3.41±1.06	3.26±0.96	3.43±1.14	P = 0.199
				1.432				9.112				2.048
HDL-C(mM)	1.61±0.34	1.52±0.49	1.69±0.32	P = 0.103	1.42±0.17	1.33±0.38	1.92±0.00	P = 0.230	1.18±0.37	1.29±0.34	1.31±0.34	P = 0.121
				2.380				25.481				2.779
lnTG	0.16±0.50	0.22±0.55	0.19±0.49	P = 0.709	0.05±0.60	0.11±0.57	-0.54±0.00	P = 0.604	0.20±0.69	0.18±0.50	0.26±0.48	P = 0.629
				0.323				9.592				0.536
TG (mM)	1.17	1.25	1.21		1.05	1.12	0.58		1.22	1.20	1.30	
ApoA-1(mg/dl)	151.86±23.78	150.69±24.82	148.72±21.16	P = 0.853	146.00±11.12	145.50±22.35	160.00±0.00	P = 0.230	134.44±28.66	139.32±24.63	147.57±27.20	P = 0.094
				0.196				21.127				3.314
ApoB(mg/dl)	97.29±28.20	95.84±30.02	88.88±26.80	P = 0.520	100.33±23.96	102.50±10.05	85.00±0.00	P = 0.687	116.48±33.29	106.74±27.52	117.37±34.39	**P** = **0.014**
				0.689				7.222				4.429
lnLp(a)	2.53±1.23	2.41±1.02	2.45±1.07	P = 0.882	2.77±1.01	2.97±1.06	2.48±0.00	P = 0.909	2.90±1.02	2.75±0.98	3.00±0.89	P = 0.590
				0.185				2.362				1.325
Lp(a)(mg/dl)	12.60	11.18	11.55	-	15.95	19.49	11.94	-	18.11	15.61	20.12	-

**Figure 2:**
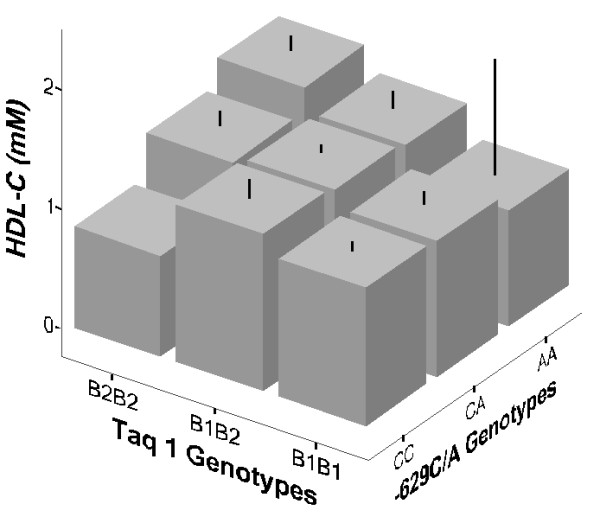
**Mean plasma HDL-C levels of the *Taq1B *and -*629C/A *composite genotypes in the control group of Chinese men.** Error bars denote 95% confidence limits.

Since there are two polymorphisms on the same *CETP* gene, we examined the combined effects of both polymorphisms on plasma lipid levels using their composite genotypes. All 9 possible combinations of genotypes are present in our population. However, two composite genotypes, *CC*/*B2B2* and *AA*/*B1B1*, were excluded from the analysis because there was only one subject in the former and two subjects in the latter although their levels are still shown in Figure 2. Significant effects of the composite genotypes on HDL-C and apoA1 in the Chinese men are observed. ANCOVA results showed that these are highest when both polymorphic sites are homozygous for the minor alleles (Figure 2).

We also carried out two-way ANCOVA to assess the effects of the two polymorphisms on HDL-C and apoA1 levels in the control group of Chinese men. When the *Taq1B* polymorphism was included in the two-way ANCOVA model with adjustment for the confounding effects of age, BMI, smoking, hypertension and diabetes mellitus, the -*629C* > *A* promoter site showed no significant association with HDL-C and apoA1 (p = 0.455 and 0.812, respectively). However, in the presence of the promoter genotypes, the *Taq1B* polymorphism remained significantly associated with HDL-C and apoA-1 (p = 0.039 and 0.009 respectively) indicating that the effects of the *Taq1B* polymorphism are independent and not due to its weak LD with the promoter polymorphism.

Moreover, we performed a fixed effect meta-analysis across all three populations (Q > 0.05 and I^2^ = 0.00). The *Taq1B* polymorphism showed highly significant association with plasma HDL-C in the combined population (p = 5.502e-06). However, -629C > A had no association with HDL-C (p = 0.0991).

### Gene-environmental interaction

**Figure 3:**
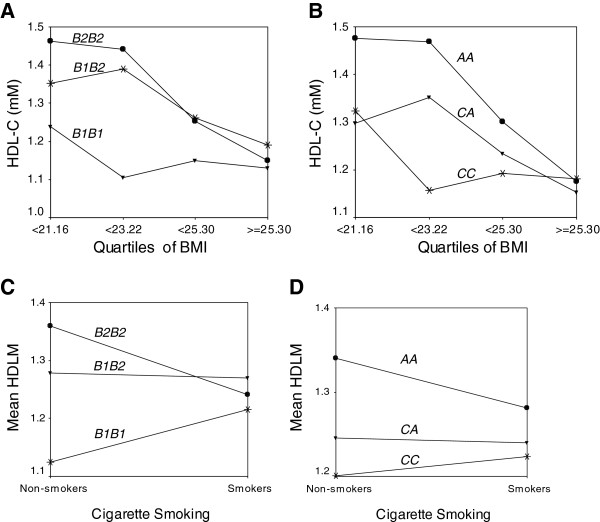
**Mean genotypic plasma HDL**-**C levels in the control group of Chinese men according to A**) **BMI quartiles and *Taq1B *genotypes**; **B**) **BMI quartiles and** -***629C*** > ***A *genotypes**; **C**) **Cigarette smoking and *Taq1B *genotypes**; **D**) **Cigarette smoking and** -***629C*** > ***A *genotypes**.

Since BMI was found to be a significant confounding factor of HDL-C and apoA1 levels, we examined the interaction of *CETP* genotypes with BMI. Figure 3A shows that the protective effect of the *B2* allele was only apparent for Chinese men subjects having BMI lower than the 50^th^ percentile, beyond which, HDL-C levels were no longer significantly different between genotypes. This was confirmed by carrying out ANCOVA separately for subjects below and above the 50^th^ percentile. The *Taq1B* polymorphism effect was only significant (p = 0.007) for the lower 2 quartiles but not the upper two (p = 0.302). The -*629C* > *A* promoter polymorphism followed a similar trend in the Chinese men (Figure 3B).

Another environmental factor which interacted with the *Taq1B* genotypes is cigarette smoking. We found significant protective effect of the *B2* allele only in non-smoking Chinese men (Figure 3C). This was also confirmed by ANCOVA performed separately for non-smokers (0.001) and smokers (p = 0.943). One should not interpret from this figure that B1B1 smokers have higher HDL-C levels as the difference in HDL-C levels between the smokers and non-smokers is not statistically significant. A similar trend was also observed for the -*629*C > A polymorphism (Figure 3D).

## Discussion

In this study we genotyped three Asian ethnic populations in Singapore for two polymorphisms of the CETP gene to determine their allele frequencies and association with CAD and plasma lipid levels. The allele frequencies of the *Taq1B* polymorphism showed marked ethnic differences. The *B1* allele is most common in the Malays but is the minor allele in the Indians. The minor *B2* allele in the Chinese and Malays are close to most Caucasian frequencies reported [38].The allele frequencies of *Taq1B* polymorphism in Indians, Malays and Chinese are consistent with the respective studies conducted previously in Sri Lankans [25], Taiwanese [27] and Singaporeans [33]. Similar to *B1*, the -*629C* allele is also the most common in the Malays (0.60) but is the minor allele in the Indians (0.30).

Among the three major ethnic groups in Singapore, the Chinese is the only group that shows a significantly lower frequency of the *B2* allele in CAD + cases compared to the controls. This finding is consistent with observation in the Caucasians [11] and suggests the association of the *B2* with some protective factors of CAD in Chinese population. Chinese subjects without the *B2* allele have 2.0 times higher risk of CAD relative to those who have at least one copy of the *B2* allele. This is independent of the effects of age, smoking, BMI, gender, hypertension, dyslipidemia and diabetes mellitus. However, this effect is not observed in the Malays and Indians. In the Taiwanese, the odds ratio for CAD was slightly higher for the *B1B1* than *B2B2* group, but no significant difference in *Taq1B* allele distribution was observed between the control and CAD groups [27]. Padmaja et al. [31] demonstrated that CETP *B1B1* of Taq1B was significantly associated with increased risk for CAD (OR 2.7; 95% CI 1.5-3.3) in the Indian population. The *B2* allele is unlikely to be a functional mutation as its position in the intron is not known to affect RNA splicing or serves any other regulatory purposes. As such, any phenotype that it is associated has to be due to its LD with another functional site that is either within the *CETP* gene or its functionally related genes nearby, such as the lecithin-cholesterol acetyl transferase gene. Dachet et al. [22] first reported the strong LD between -*629C* > *A* and *Taq1B* polymorphisms. Later many more alleles in the *CETP* gene [39-41] were also reported to be in LD with the *Taq1B* site. In the Singaporean population, weak LD between *Taq1B* and -*629C* > *A* was consistently observed in all three ethnic groups, although to varying extent. It is highest in the Malays, followed by Chinese and Indians. Wu et al. [23] observed a LD between Taq1B and -629C > A polymorphic sites in the Chinese population from China.

The effect of the *Taq1B* intronic polymorphism in this study could not be generalized across ethnic groups and genders. This is expected since the magnitude of its effects is dependent upon the strength of its LD with a functional site. Among the lipid traits that we have studied, the *B2* allele is most evidently associated with raised levels of protective factors such as HDL-C and its associated apoA1. This trend of association was consistent in all ethnic groups and genders although statistical significance was attained only in the Chinese men. Significant association of the *B2* allele with elevated HDL-C was also reported for the Framingham population [11], Chinese population [23], Iranian population [19,20] and Tunisian population [9]. In their studies, the protective effects of the *B2* allele on the development of CAD were observed in association with increased HDL-C and decreased CETP activity. The association of the *B2* allele with higher plasma HDL-C and / or apoA1 has been consistently observed in many other studies [15,24,27,28,33,42] as well. However, Rahimi et al. [14] indicated that the CETP*B1* allele is associated with increased risk of CAD and type 2 diabetes mellitus independent of plasma HDL-C level in the Iranian population.

In this study, we have shown that the association of the *Taq1B* site with HDL-C and apoA1 levels remains independent of the effects of the promoter -*629C* > *A*. Both sites have similar effects on apoA1. We asked the question of whether the -*629C* > *A* site is the functional mutation while *Taq1B* was merely showing significant association with plasma lipid levels as a result of LD. We concluded that this may not be the case. Firstly, a functional mutation is likely to have its effect observed across all ethnic groups and genders. However, we observed effects of the promoter polymorphism only in Chinese men. Although we cannot be conclusive that the -*629C* > *A* polymorphism is not a functional mutation since other genes and environmental factors could have masked its moderate effect, its ethnic-and gender-specific effects nevertheless suggests the unlikelihood of this being so. Secondly, the strength of association with HDL-C and apoA1 levels was consistently higher for the *Taq1B* site than the promoter site. This should not be the case if the -*629C* > *A* site is functional and *Taq1B* site its marker. Moreover, in the two-way ANCOVA, there was no effect of the promoter polymorphism when the *Taq1B* site was included in the model. We postulated that there could be other functional sites within or near the *CETP* gene that the *Taq1B* site is in stronger LD with than the promoter site. Both *Taq1B* and -629C > A polymorphisms could possibly have independent effects on HDL-C and apoA1 levels and hence they do not show significant effects individually when they are included in the same 2-way ANCOVA model. In the Turkish population, it was found that *CETP* -*629C* > *A* polymorphism was not associated with CAD [43]. As with any complex traits, the genotypic effect is usually confounded by environmental factors. In this study, we found significant gene-environmental interaction of the *Taq1B* polymorphism with BMI and smoking. The protective effect of the *B2* allele was only observed in subjects having BMI <23 and in non-smokers. This is consistent with other studies that examined the confounding effects of BMI [21,28,38] and smoking [15,25,38,44].

We concluded that, i) the absence of the *B2* allele was associated with CAD in the Chinese, ii) the minor alleles of the two polymorphisms were significantly associated with higher plasma HDL-C levels in the Chinese men (*B2*), iii) the effects of the polymorphisms were significant only in non-smoking subjects with BMI up to the 50^th^ percentile, iv) The effect of the *Taq1B* polymorphism is not entirely dependent on the -*629*C/A site but that it could be in LD with some other functional sites.

## Competing interests

The authors declare that they have no competing interests.

## Authors’ contributions

YL carried out genotyping. NT and CKH carried out data analysis. HL assisted in the genotyping. NS was responsible for subject recruitment. HY and CKH contributed to the conception and design of this study. NT and CKH revised the manuscript. All authors read and approved the final manuscript.
